# Lessons Learned to Date on COVID-19 Hyperinflammatory Syndrome: Considerations for Interventions to Mitigate SARS-CoV-2 Viral Infection and Detrimental Hyperinflammation

**DOI:** 10.3389/fimmu.2020.01131

**Published:** 2020-05-29

**Authors:** Marco Cardone, Masahide Yano, Amy S. Rosenberg, Montserrat Puig

**Affiliations:** Laboratory of Immunology, Division of Biotechnology Review and Research III (DBRR III), Office of Biotechnology Products (OBP), Office of Pharmaceutical Quality (OPQ), Center for Drug Evaluation and Research (CDER), FDA, Silver Spring, MD, United States

**Keywords:** coronavirus, 2019-nCoV, SARS-CoV-2, antiviral immune response, severe COVID-19, hyperinflammation, cytokine release syndrome, treatment strategies

## Abstract

The first case of human transmission of SARS-CoV-2 was reported in China in December 2019. A few months later, this viral infection had spread worldwide and became a pandemic. The disease caused by SARS-CoV-2, termed COVID-19, is multifactorial and associated with both specific antiviral as well as inflammatory responses, the extent of which may determine why some individuals are asymptomatic while others develop serious complications. Here we review possible life-threating immune events that can occur during disease progression to uncover key factors behind COVID-19 severity and provide suggestions for interventions with repurposed drugs in well-controlled and randomized clinical trials. These drugs include therapeutics with potential to inhibit SARS-CoV-2 entry into host cells such as serine protease inhibitors of the cellular protease TMPS2 and drugs targeting the renin-angiotensin system; antivirals with potential to block SARS-CoV-2 replication or factors that could boost the antiviral response; monoclonal antibodies targeting pro-inflammatory cytokines that drive the hyperinflammatory response during COVID-19 progression toward the severe stage and therapeutics that could ameliorate the function of the lungs. Furthermore, in order to help make more informed decisions on the timing of the intervention with the drugs listed in this review, we have grouped these therapeutics according to the stage of COVID-19 progression that we considered most appropriate for their mechanism of action.

## Introduction

Since the first cases reported from Wuhan, China, in December 2019, the Severe Acute Respiratory Syndrome Coronavirus 2 (SARS-CoV-2), which was initially referred to as 2019-nCoV, has spread worldwide as not seen since the influenza pandemic in 1918. Global changes in social behavior including the ability to travel internationally, played an important role in the spread of the disease known as COVID-19, to more than 187 countries and regions, as of May 7th, 2020. Although SARS-CoV-2 belongs to the coronavirus family, the epidemiology of COVID-19 differs from that of previously emerged, SARS-CoV- and MERS-CoV-induced diseases in its greater ability to be transmitted among communities, resulting in a larger number of patients infected. Although the frequency of infections that progress to severe disease is less for SARS-CoV-2 than for either SARS-CoV or MERS-CoV, the higher number of overall infections has resulted in a greater number of patients with severe acute respiratory symptoms that require clinical intensive care. Understanding the characteristics of the viral infection, as well as the host response to the virus, is critical to making informed decisions regarding the most effective strategy to combat the disease in its stage specific manifestations.

The severity of COVID-19 has been associated with progression to severe disease if virus burden is not properly controlled at the early stage of infection (1). Increasing evidence shows that the probability of progressing toward severe disease is greater in men than women and increases with age, with the most vulnerable individuals being older adults and those with at least one pre-existing condition diagnosed before the infection. Comorbidities associated with COVID-19 severity are hypertension, diabetes, cardiovascular disease, chronic kidney disease and chronic obstructive pulmonary disease (COPD) (2, 3).

The early stage of SARS-CoV-2 infection is characterized by mild or absent symptoms. Asymptomatic individuals can still infect others, justifying the need for social distancing as a preventive measure, until safe and effective prophylactic and therapeutic options become available. Common mild symptoms of the early stage of infection are fever, dry cough, myalgia and fatigue. Less common are sputum production, headache, hemoptysis and diarrhea. Clinical laboratory signs include lymphopenia, which occurs 4–8 days after disease onset, with circulating lymphocyte count typically < 1.0 × 10^9^/L. During the transition toward the severe stage, symptoms such as dyspnea (median time for appearance is approximately 8 days from the onset of symptoms) and hypoxia develop. This progression can also be associated with abnormal lung computed tomography (CT) scans, neutrophilia, increased prothrombin time and increased D-dimer. Finally, the severe stage of COVID-19 disease manifests with Acute Respiratory Distress Syndrome (ARDS), which typically appears by day 9 from the onset of illness and accompanied by severe lung inflammation and damage. Appearance of these severe symptoms is often associated with increased levels of C-reactive protein (CRP), lactate dehydrogenase (LDH), D-dimer, ferritin, troponin, N-terminal pro-brain-type natriuretic peptide, and IL-6. At this stage, patients often require intensive care unit (ICU) admission and life support with mechanical ventilation. As the disease worsens, respiratory failure persists, despite mechanical ventilation, and diffuse vascular complications and myocarditis may develop (2–13). In such progressive cases, death occurs by day 14 from the appearance of the first symptoms. Complications at the severe stage of disease are the leading cause of death among critically ill patients with SARS-CoV-2 infection and several studies have reported the association of these complications with virus-induced hyperinflammation (4, 7, 11), similar to that seen in SARS-CoV and MERS-CoV infections (14–16).

The hyperinflammation observed in adult patients with severe COVID-19 (both in ICU and non-ICU care) is characterized by increased plasma levels of the following: pro- and anti-inflammatory cytokines (IL-1β, IL-7, IL-8, IL-9, IL-10, IFN-γ, TNF); chemokines known for their ability to attract neutrophils, myeloid cells, T lymphocytes, and NK cells to the site of infection and inflammation (MCP1, MIP1A, MIP1B); and growth factors (G-CSF, GM-CSF). In addition, levels of IL-2, IL-7, IL-10, G-CSF, IP10, MCP1, MIP1A, and TNF were found to be higher in ICU patients as compared to those not admitted to the ICU (7). Furthermore, elevated serum levels of IL-6 have been reported to be significantly associated with death among severe COVID-19 cases (11). Based on these clinical parameters, Mehta et al. (17) suggested that the immunologic profile of disease in severe COVID-19 patients resembles that of the cytokine release syndrome (CRS), and secondary hemophagocytic lymphohistiocytosis (sHLH), also recognized as the macrophage activation syndrome (MAS) (18).

Of note, COVID-19 is a complex disease involving both cellular and humoral immunity. Acute antibody responses against SARS-CoV-2 nucleoprotein and spike protein epitopes (IgM and IgG) have been observed in COVID-19 patients (19). In addition, neutralizing antibodies, mostly against the viral S-protein, have been detected in convalescent patients (20, 21). These observations indicate that humoral responses are mounted rapidly in COVID-19 patients and could play an important role in protection. Nevertheless, antibody-dependent enhancement has been proposed as a mechanism to exacerbate SARS-CoV-2 infection (22). Thus, the different potential roles of antibodies in COVID-19 are still under debate. However, the principal aim of this review is to dissect immunological events that lead to cytokine release syndrome and COVID-19 severity.

Below, we review several possible immunological events underlying the virus-induced shift from protective antiviral immunity into a hyperinflammatory response leading to the life-threatening inflammation observed in critically ill COVID-19 patients. Due to the lack of readily accessible animal models and experimental data for COVID-19, these immune events are modeled and illustrated by integrating information from currently available reports on COVID-19 with information from the scientific literature pertaining to SARS-CoV and MERS-CoV infections, as well as mechanisms of CRS. This approach was taken to identify critical immunopathologic factors and biomarkers of such factors in the evolution of SARS-CoV-2 infection, that could be preventively and/or therapeutically targeted in clinical trials of currently available antivirals, immunomodulators and other drugs already approved for other infections and inflammatory diseases (repurposing use), while new vaccines and specific therapies become available.

## Early Stage of Infection: Determinants of An Effective Antiviral Response Vs. Host Immune Dysregulation

### Viral Entry

SARS-CoV-2 can enter the human body by inhaled respiratory aerosols and droplets containing viral particles, and by contact with contaminated surfaces, although the importance of this second possible mode of infection has not been established (5, 7, 23, 24). SARS-CoV-2 particles have been shown to be stable and remain infectious for hours in aerosols or even days on surfaces (25).

**Figure 1:**
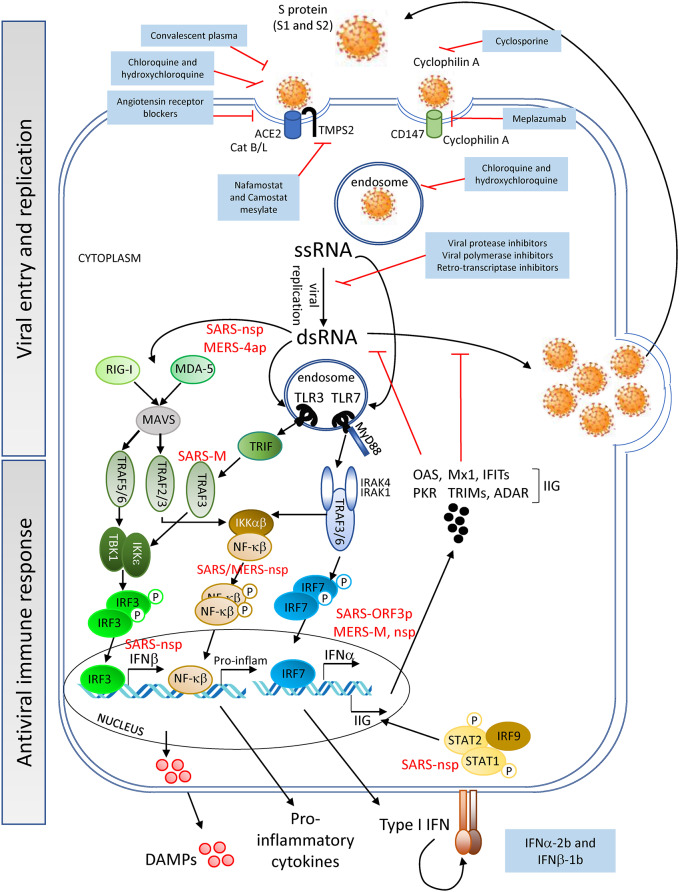
Viral entry and replication, and host antiviral immune response—SARS-CoV-2 (Spike (S)-protein) recognizes cell surface proteins (ACE2, TMPS2, and CD147) which facilitate the endocytosis of the virus particle. Viral genome (ssRNA) exits the endosomal vesicles and starts the replication cycle generating dsRNA intermediates, protein translation, encapsidation and generation of new viral particles. New virions can subsequently infect neighboring cells. DAMPs are being released by the dying cells into the extracellular space. Innate immune receptors such as TLR3, RIG-I and MDA-5 or TLR7 can sense viral RNA (dsRNA or ssRNA, respectively) and initiate a signaling cascade for the production of IFNα/β and pro-inflammatory cytokines. Interferon-induced genes (IIG) will be expressed as a result of this type I IFN feedback loop, blocking viral replication. SARS-CoV and MERS-CoV structural and non-structural proteins can interfere with the cells' innate immune response [see proteins in red; for further information refer also to the Kindler et al. (29)], delaying the production of sufficient levels of antiviral cytokines to prevent control of viral replication at the early stages. SARS-CoV-2 might utilize similar evasion mechanisms, although no data have yet been reported. Pharmacological interventions (blue-background boxes) are being proposed to block both the entry and genomic replication of SARS-CoV-2, as well as to boost the innate immune response.

SARS-CoV-2 binds to angiotensin converting enzyme 2 (ACE2) expressed on cell surfaces via its spike (S) protein and penetrates host cells on activation and catalytic activity of the cellular transmembrane protease serine 2 (TMPS2) (encoded by the gene *TMPRSS2*) (26–28) (Figure 1). ACE2 is expressed on cells of numerous tissues including the following: lung alveoli; nasal, oral, dermal and kidney epithelia; smooth muscle; and endothelial cells of vessels in the gastrointestinal tract as well as in arterial and venous vessels (30, 31). The interaction of the viral S-protein with the ACE2 cellular receptor may result in the dysregulation of the renin-angiotensin system, diminishing the levels of ACE2 and increasing those of angiotensin II [found elevated even in severe CRS (32)], potentially contributing to the impairment of vessel and lung homeostasis. An ACE2 KO mouse model, in which animals were challenged with acid aspiration or sepsis (33), showed that the loss of ACE2 activity lead to increased vascular permeability, lung edema and inflammation due to neutrophil influx. Similarly, these disruptive events may contribute to the pathology in patients with severe SARS-CoV-2 infection and could explain the development of lung injury in critically ill COVID-19 patients. In support of this hypothesis, infiltrating neutrophils and pro-inflammatory macrophages have been found in the lungs of patients who died of SARS-CoV infection (34, 35). Additionally, patients with SARS-CoV-2 and ARDS have been reported to develop neutrophilia, suggesting that neutrophils contribute to lung inflammation and damage in severe COVID-19 disease (10, 11). These events may synergize with the host immune response to the virus infection discussed below.

Interestingly, though ACE2 was not detected in lymphocytes within the lymphatic organs (30), it was detected in lymphocytes infiltrating the oral mucosa (31). However, the latter study only evaluated ACE2 expression at the transcript level, and protein expression of ACE2 on lymphocytes thus needs to be confirmed. Nevertheless, from the detection of SARS-CoV-2 particles and genomes in lymphocytes, along with the persistent lymphopenia observed in patients with moderate/severe COVID-19 (36), arose the hypothesis that the infection of these cells might be mediated through an alternative receptor, CD147 (37) (Figure 1). CD147 is a transmembrane glycoprotein expressed in tumors, inflamed tissues and pathogen-infected cells, with a role in the regulation of cytokine secretion and leukocyte chemotaxis (38, 39). Cyclophilin A, a natural ligand for CD147, has been shown to facilitate replication of viruses including coronaviruses (40). Although from a study with a small cohort of COVID-19 patients, clinical data showed an association of CD147 blockade by meplazumab, with an improvement in lymphocyte counts, viremia and chest CT scan (37). However, due to the limited evidence, further studies are needed to confirm the role of CD147 in SARS-CoV-2 entry.

### Viral Replication and Innate Immune Sensors

**Figure 2:**
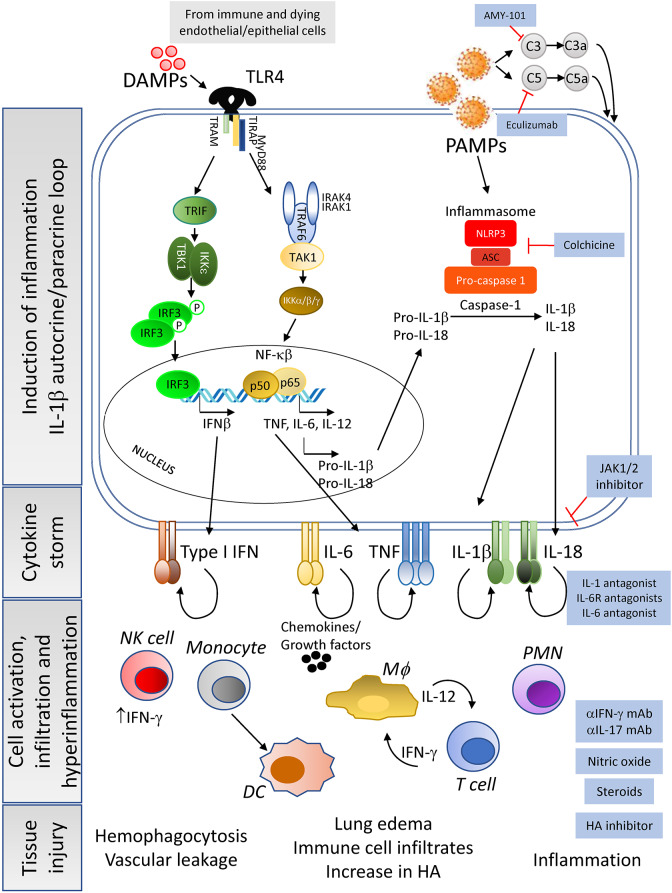
Cause and consequences of the CRS—Constant exposure to DAMPs from dying infected cells and to high viral titers (pathogen associated molecular patterns, PAMPs) lead to the enhancement of pro-inflammatory cytokine pathways in immune cells and tissue resident cells. In addition, complement activation leads to macrophages activation and cytokine release. Of importance is the induction of the IL-1β autocrine loop, involving the activation of the inflammasome complex that results in high levels of this cytokine being secreted to the extracellular space. Release of IL-1β and subsequent engagement with its receptor will enhance the production of other pro-inflammatory cytokines by the activated cells, leading to a massive release of cytokines, chemokines and growth factors. This cytokine storm creates an inflammatory microenvironment in the tissue, already experiencing elevated inflammation due to the dysregulation of angiotensin II levels, that will feedback into hyperactivation of resident immune cells, as well as mobilization of peripheral immune cells into the tissue. The end result of the dysregulation of the host immune response will be tissue damage and organ failure with the possibility of patient death as severity increases. Pharmacological interventions (blue-background boxes) are being proposed to control or manage the tissue and systemic hyperinflammation detected in moderate and severe cases of COVID-19, by agents that can block the binding of cytokines to their receptors as well as drugs that inhibit the synthesis of hyaluronic acid to prevent pulmonary edema.

Once inside the intracellular space of the host cell, the SARS-CoV-2 positive single strand (ss) RNA genome initiates its replication using self and host proteins (Figure 1). At this point, both genomic ssRNA and double stranded (ds) RNA intermediate molecules can be recognized by the host immune system. The innate immune sensors capable of being activated through the recognition of foreign RNA are TLR3, RIG-I and MDA5 for dsRNA, and TLR7 and TLR8 for ssRNA. Their activation typically triggers the antiviral machinery of cells, starting with the generation of type I IFN (Figure 1). No data have been published so far to elucidate the interaction of SARS-CoV-2 proteins with antiviral mediators of the innate immune system of the host. However, if SARS-CoV-2 uses mechanisms similar to those employed by SARS-CoV or MERS-CoV to evade the early steps of the host innate antiviral response (29, 41–43), it is probable that SARS-CoV-2 proteins interfere with the activation of the type I IFN pathway. If so, both structural and non-structural proteins of the novel coronavirus could inhibit critical steps of the type I IFN pathway (Figure 1), thereby delaying the production of type I IFN (in both magnitude and time), resulting in an altered antiviral immune response. Evidence that SARS-CoV-2 impairs expression of type I and III IFN genes has been shown *in vitro* (in primary human lung epithelium and alveolar cell lines), in a SARS-CoV-2 animal model, and in lung autopsies and serum from COVID-19 patients (44). Thus, a compromised RNA-specific innate immune response, at the beginning of the infection, could compromise control of virus replication, leading to a dramatic increase in the viral titer and the number of infected cells, as has indeed been observed in a mouse model of SARS-CoV infection (45). Epithelial and endothelial cells with actively replicating virus will eventually become apoptotic and die, further contributing to tissue inflammation by releasing high levels of IL-1β (upon NALRP3 inflammasome activation) and danger molecules or damage-associated molecular patterns (DAMPs) into the extracellular environment. DAMPs will be subsequently recognized by innate immune receptors on resident immune cells such as alveolar macrophages, enhancing the inflammatory autocrine loop of IL-1β and type I IFNs (Figure 2). Of note, a novel linage of lung-resident macrophages, named nerve and airway-associated macrophages or NAMs, was recently described (46). NAMs, unlike alveolar macrophages, exert immune suppressive functions and thus could contribute to maintain the homeostasis of the lung during pathogen infections. The potential role in lung protection is currently under investigation.

COVID-19 related inflammatory responses could also be induced by the dysregulation of the complement system, a critical component of the host innate immunity. Although it is aimed to prevent viral replication, excessive activation of complement components such as C3, C3a, C5, C5a, and mannose binding lectin-associated serine protease (MASP2), possibly by viral proteins, has been associated with increased inflammation both in SARS-CoV and SARS-CoV-2 infections (47–49). C3a and C5a overexpression can activate alveolar macrophages through their respective receptors leading to the release of pro-inflammatory cytokines such as IL-6 (Figure 2). This lack of immune control can exacerbate respiratory and vascular disease (48–50).

## Transition and Progression Toward The Severe Stage of Infection

Although it is not yet clear how CRS develops downstream of the initial immune response to SARS-CoV-2, the induction of cytokines by viral RNA activation of the innate immune system, as demonstrated for influenza (51), and the release of DAMPs by apoptotic and necrotic cells (52), have been proposed as possible triggers. Alarmins such as the high mobility group box-1 (HMGB-1), a nuclear protein abundantly released by necrotic cells or actively secreted by macrophages and NK cells, as well as by infected cells, have been shown to contribute to the overproduction of IL-1β following their activation of the cellular inflammasome, triggered by innate immune sensors such as TLR4 and the NF-κβ pathway during the development of MAS (53). Moreover, ferritin, which is a biomarker in CRS and found elevated in severe SARS-CoV-2 patients, has also been proposed to act as a DAMP in sHLH (52), a condition thought to be pathogenically similar to the cytokine storm in patients with severe COVID-19.

Downstream, the increased levels of IL-1β could then activate, via autocrine and paracrine recognition, innate immune cells expressing IL-1R, including macrophages and NK cells, thus amplifying inflammation with the release of high levels of the pro-inflammatory cytokines IL-6, IL-18, TNF, and IL-1β by macrophages, and of IFN-γ by NK cells (Figure 2). IL-6 and IFN-γ, which are present at high levels in the plasma of severe COVID-19 patients, are hallmarks of CRS (32). Further supporting the similarity of HLH and severe COVID-19 infection, increased levels of NK-produced IFN-γ have been recognized as a driver of HLH disease (52). IL-6, in turn, has been shown to promote severe CRS by inducing vascular dysfunction, including vascular leakage (32). In addition, tissue release of IL-1β and TNF by infected cells has been shown to increase levels of hyaluronan (HA) synthase 2, and consequently Hyaluronic Acid (HA) (54). HA can absorb water in high quantity (55) and may be contributing to the observed accumulation of fluid in the lungs of COVID-19 patients with ARDS, thus further compromising respiratory function. Indeed, chest x-rays, particularly of severely ill patients, revealed ground-glass opacities now considered pathognomonic of severe CoV infections (2, 4, 5, 7, 10). However, it remains to be confirmed whether these abnormalities are facilitated by HA and, if so, whether the use of Hymecromone (4-Methylumbelliferone), an inhibitor of HA-synthase-2 could improve the outcome of COVID-19 disease, as suggested by Shi et al. (55).

The immunological events described above create a cytokine- and chemokine-mediated hyperinflammatory environment in the epithelium of the lungs with the potential to recruit and hyperactivate T cells that, in turn, could contribute to the inflammatory damage of the tissue, while mounting virus specific immune responses. Tissue infiltration of T cells could also be facilitated by the upregulation of adhesion molecules by lung endothelial cells.

However, the fact that most patients with COVID-19 develop lymphopenia 4 days after the onset of symptoms led to the consideration of the mechanisms by which T cells were contributing to the detrimental inflammation induced by SARS-CoV-2. While the lymphopenia *per-se* is of unclear origin, two hypotheses are considered: infection and killing of the lymphocytes; or tissue margination/infiltration. In the latter scenario, T cells could contribute to the cytokine storm and tissue damage at the infection site. Indeed, histological examination of the organs of a 50-year-old male patient who died of pulmonary edema, ARDS and cardiac arrest 14 days after symptoms onset, showed infiltration of lymphocytes in both lungs, as well as liver injury and a mild inflammation of the heart tissue due to infiltration of mononuclear cells. Furthermore, his circulating CD4^+^ and CD8^+^ T cells, though lower in number than normal, were found to be hyperactivated, with CD4^+^ cells showing a pro-inflammatory Th17 phenotype (likely promoted by IL-1β and IL-6) and highly cytotoxic CD8^+^ T lymphocytes (4). However, it should also be noted that this patient was treated with numerous therapeutics that could confound this interpretation and therefore, it is not clear if T cell hyperactivation and lung infiltration by lymphocytes in this severe case of COVID-19 were caused by the virus, the therapeutic regimen, which included type I IFN, or the combination of the two. Regardless of the mechanism of loss of such cells in blood, two reports (8, 13, 56) indicated that circulating T and NK cells in COVID-19 patients acquire an exhausted phenotype, which became more prominent during disease progression, as it was more evident in ICU admitted patients. As markers of cell exhaustion, these studies reported the upregulation of inhibitory molecules such as NKG2A, PD-1 and TIM3 on the cell surface, as well as a reduced ability to produce pro-inflammatory cytokines (IL-2, IFN-γ, TNF) and cytotoxic factors in both T and NK cells. Reduced IFN-γ expression in CD4^+^ T cells has also been reported by Chen et al. (57), particularly in severe COVID-19 cases. However, the contribution to detrimental inflammation by T lymphocytes and NK cells likely occurred in COVID-19 patients prior to these cells becoming dysfunctional. Exhaustion, indeed, is a state in which cells show dysfunctionality after being fully active, including in their capacity to make pro-inflammatory cytokines such as IFN-γ. Hyperactivation and subsequent dysfunction of effector T cells during the progression of SARS-CoV-2 infection could also be driven by the decrease in CD4^+^ regulatory T cells (key players in protection from tissue damage by restraining hyperinflammation) observed in COVID-19 patients, especially in those progressing to severe disease (57, 58). Therefore, a more in-depth characterization of CD4^+^ and CD8^+^ T cells at the early and severe stages of the disease, possibly from the same individuals, is needed. Moreover, whether a poor prognosis in seriously ill patients is associated with the acquisition by lymphocytes of an exhausted phenotype, and whether therapeutic interventions to prevent or reverse T cell exhaustion can safely facilitate the clearance of SARS-CoV-2, perhaps in the context of therapeutics to diminish the hyperinflammatory milieu or by restoring the immune balance through the enhancement of regulatory T cell (Treg) number and activity, also need critical investigation.

## Considerations for Treatment Strategies Currently Applied To COVID-19

Given the time needed to generate a SARS-CoV-2 vaccine and in the absence of specific treatments for COVID-19, the medical community and government authorities have focused their attention on drugs already available or under development that could ameliorate the condition of patients with this infection. The rationale for considering clinical trials to assess repurposing of currently available therapeutics, including antivirals, antimalarial drugs or medications used to treat inflammatory conditions derived from their efficacy in diseases that share some clinical features with COVID-19. Information on the possible mechanism of action of several of these drugs in coronavirus infections have been captured in literature reports, including a recent review by McCreary and Pogue (59). Results from COVID-19 patients treated with repurposed drugs are increasingly being reported for both monotherapies and combination therapies. The design of these studies, however, has not yet allowed for the establishment of recommended clinical practices for COVID-19 because (1) data often originate from clinical observations from small non-randomized studies from a single center, (2) most studies have a lack of adequate control arms, (3) a lack of standardized reporting criteria, and/or (4) results are derived from a heterogeneous patient population in which drugs are switched during the course of the disease to other drugs for compassionate reasons.

Taking this into account, in this review we have summarized the relevant aspects of the pathogenesis of SARS-CoV-2 infection and highlighted important immunological events that may drive the switch of the host's immune response against the virus, from protective (antiviral) to pathogenic (hyperinflammatory), during disease progression. This is to provide basic immunological knowledge of the clinical stages of COVID-19 disease in order to help make more informed decisions about the type of treatment and the timing of the intervention to be evaluated in clinical trials.

In Supplementary Table we have listed available and potential therapeutics that have been or could be considered for entry into clinical trials to assess their possible repurposing for COVID-19. The list has been compiled with drugs that medical experts around the world are currently evaluating for SARS-CoV-2 infection plus therapeutics we have entered based on their potential to act against key players in COVID-19, highlighted in this review (see also Figures 1, 2). The table also includes (1) information on the mechanism of action of these drugs and the disease/s for which they were originally approved and/or designed, (2) a link to *ClinicalTrials.gov* (Trial Progress) where it is possible to monitor the progression of the clinical studies using these products in COVID-19 (if clinical trials were registered by the time of submission of this review), and (3) indications of the possible mechanism of action of these therapeutics in COVID-19. In addition, to help choose the timing of intervention, all the drugs listed in the table have been grouped according to the stage of disease progression that we considered most appropriate for their mechanism of action (see also Figures 1, 2).

## Conclusion

The big challenge to overcome in the fight against COVID-19 is to rapidly identify safe and effective therapies that can control the detrimental inflammation caused by SARS-CoV-2 without compromising protective antiviral immune responses of the patients (60). Therefore, we emphasize that the drugs and the timing of the intervention that we are suggesting are only for the purpose of helping make more informed decisions among available options for clinical investigation and development, and may not be safe and/or effective for all patients, especially when the risk related to both the possible side effects of the drug and to the pre-existing condition of the patient may outweigh the potential benefit. Until vaccines and targeted drugs for COVID-19 are available, there may be a need to intervene with personalized therapeutic approaches. We are learning day after day, that patients may be affected by SARS-CoV-2 differently and that many factors influence the outcome of the disease. Thus, due to the rapidly changing landscape of clinical trials for COVID-19, we caution the reader that some of the information listed in Supplementary Table, current at the time of submission of the review, may have changed or withdrawn in the interim till this publication. Updates on vaccines and therapies under study for COVID-19 can also be obtained from sources such as BioCentury (https://www.biocentury.com/clinical-vaccines-and-therapies). Finally, we believe that the information summarized in this review provides the starting point for a more elaborate immunologic dissection of COVID-19, from which new therapeutic interventions may emerge for evaluation in the context of well-controlled and randomized clinical trials, clearly critical for obtaining data to determine safety and effectiveness of clinical strategies to vanquish SARS-CoV-2.

## Author Contributions

MC and MP wrote the manuscript. MC, MY, and MP performed the literature review and data collection, and prepared the Supplementary Table. MP designed the Figures. MC, AR, and MP revised the manuscript.

## Conflict of Interest

The authors declare that the research was conducted in the absence of any commercial or financial relationships that could be construed as a potential conflict of interest.
