# Supplementary material for: Lessons Learned to Date on COVID-19 Hyperinflammatory Syndrome: Considerations for Interventions to Mitigate SARS-CoV-2 Viral Infection and Detrimental Hyperinflammation
Source: Front Immunol. 2020 May 29;11:1131. doi: 10.3389/fimmu.2020.01131 (PMC7272717; doi:10.3389/fimmu.2020.01131)
Supplement: Supplementary file 1 [file Table_1.pdf]

Supplementary Table - Summary of therapies that are being investigated and/or considered for the COVID-19 treatment

COVID-19 progression	Drug name/Therapeutics	Mechanism of action/Potential role in COVID-19	Drug status	Clinical trials	References
**Innate/Adaptive Immune Activator**	**Bacillus Calmette–Guérin (BCG) Vaccine**	• Adjuvant with immunomodulatory properties<br>• Enhances activation of the innate and adaptive immune system and has been shown to protect not only from *M. tuberculosis*, but also from a variety of unrelated pathogens<br><br>Observations in countries with universal BCG vaccination policy suggest that BCG may reduce COVID-19 morbidity and mortality	Approved for prevention of tuberculosis (in persons not previously infected with *M. tuberculosis* who are at high risk for exposure)	Trial Progress	(61, 62)
**Inhibition of Viral Entry**<br><br>**(See also Figure 1)**	**Nafamostat**<br><br>**and**<br><br>**Camostat Mesylate**	• Synthetic serine protease inhibitors of transmembrane protease serine 2 (TMPS2), a cell surface protease encoded by the gene *TMPRSS2*<br><br>The entry of host cells by SARS-CoV-2 implies the interaction and proteolysis of viral S-protein through the cellular receptor ACE2 and the cellular protease TMPS2, respectively. This is to ensure the fusion of SARS-CoV-2 and host cell membranes. *In vitro* data using mammalian cell lines (including lung cell lines) suggest that these serine protease inhibitors impair S-protein-initiated membrane fusion by blocking TMPS2 activity	Not FDA Approved<br><br>Approved in Japan as an anticoagulant therapy for patients undergoing continuous renal replacement therapy due to acute kidney injury	Trial Progress (with Nafamostat)<br><br>Trial Progress (with Camostat Mesylate)	(26, 63)
	**Angiotensin Receptor Blockers (ARBs)**<br><br>**and**<br><br>**Angiotensin Converting Enzyme Inhibitors (ACEIs)**	• Drugs targeting the renin-angiotensin system<br>• ARBs prevent angiotensin II from binding to its receptor<br>• ARBs include recombinant ACE2, Ang 1-7 peptides, angiotensin II receptor inhibitors, and potentially aldosterone synthase inhibitors<br>• ACEIs prevent the conversion of angiotensin I into angiotensin II<br><br>Recombinant human soluble ACE2 showed inhibition of SARS-CoV-2 infection on engineered human organoids (capillary and kidney) *in vitro*. ARBs and ACEIs may have a beneficial potential in COVID-19 as they are used to treat comorbidities associated with the high risk of developing severe disease. The increase of ACE2 expression by these inhibitors has not been proved to facilitate viral infection	Approved for the treatment of hypertension, heart failure or diabetic nephropathy	Trial Progress	(64-67)
	**Hydroxychloroquine/ Chloroquine**	• Antimalarial drugs with potential to increase endosomal pH and inhibit the entry of a broad spectrum of viruses into host cells<br>• Have anti-inflammatory properties<br><br>Inhibit SARS-CoV-2 infection in Vero cells (a monkey kidney epithelial cell line), possibly by altering the endosomal pH and affecting the interaction of viral S-protein with ACE2, as shown for SARS-CoV. Preliminary data from China seem to indicate that chloroquine may improve the outcome of COVID-19 patients. Chloroquine anti-inflammatory properties may prevent hyperinflammation leading to severe COVID-19	Approved for the prophylaxis/treatment of uncomplicated malaria, rheumatoid arthritis (RA), and systemic lupus	Trials Progress	(68-72)
	**Meplazumab**	• Humanized mAb against CD147, a transmembrane glycoprotein expressed in leukocytes, as well as epithelial and endothelial cells<br><br>Reduces SARS-CoV-2 replication in Vero cells, likely by blocking the interaction between S-protein on SARS-CoV-2 and CD147. A preliminary clinical report showed that the use of meplazumab appeared to improve the outcome of COVID-19 patients	Not FDA Approved	Trial Progress	(37)

COVID-19 progression	Drug name/Therapeutics	Mechanism of action/Potential role in COVID-19	Drug status	Clinical trials	References
**Inhibition of Viral Replication**<br><br>**(See also Figure 1)**	**Cyclosporine and Tacrolimus**	• Small molecules with anti-inflammatory properties<br>• Act as calcineurin inhibitors by binding to cyclophilins or 12-kD FK-binding protein, respectively, and blocking the activation of the calcineurin/NFAT pathway, which plays an essential role in the activation of immune cells, in particular T lymphocytes, through the induction of several pro-inflammatory cytokines<br><br>SARS-CoV NSP1 can enhance the activation of the calcineurin/NFAT pathway and the expression of IL-2 *in vitro*. Moreover, cyclosporine was shown to inhibit the *in vitro* replication of several coronaviruses including SARS-CoV by blocking the calcineurin pathway. Therefore, calcineurin inhibitors could potentially limit viral replication and/or reduce disease complications due to hyperinflammation in COVID-19 patients	Approved for immunosuppression during organ transplantation. Cyclosporine is also approved for rheumatoid arthritis (RA), psoriasis, nephrotic syndrome due to glomerular disease	No registered trials to date with cyclosporine<br><br>Trial Progress (with Tacrolimus)	(73-75)
	**Favipiravir**	• Antiviral drug (modified pyrazine analogue)<br>• Acts as a RNA-dependent RNA polymerase (RdRp) viral enzyme inhibitor, with potential to block viral genome replication of a broad spectrum of RNA viruses<br><br>Reduces SARS-CoV-2 infection *in vitro* when administered at high concentrations to Vero E6 cells	Not FDA Approved<br><br>Approved for influenza in Japan and China	Trial Progress	(68, 76)
	**Remdesivir**	• Antiviral drug (adenosine triphosphate analogue), in clinical development as a potential treatment for Ebola infection<br>• Inhibits the viral RNA polymerase function of a broad spectrum of viruses. It incorporates into the viral RNA and induces its early termination during the replication of the viral genome<br><br>Efficiently controls SARS-CoV-2 infection *in vitro* when administered at low micromolar concentration to host cells, as observed for SARS-CoV and MERS-CoV. *In vivo*, showed a protective effect against SARS-CoV infection in mice. Compassionate use of remdesivir has shown improvements in the outcome of COVID-19 patients	Not FDA Approved	Trials Progress	(68, 77-81)
	**Sirolimus**	• Inhibits viral replication, possibly at the transcriptional level, by targeting mammalian target of rapamycin (mTOR)<br>• Inhibits T lymphocyte activation/proliferation that occurs in response to antigenic and cytokine (IL-2, IL-4, and IL-15) stimulation, although this might not be the mechanism of action involved in impairing viral replication, at least for HCV<br><br>Inhibits replication of MERS-CoV *in vitro* in the human hepatocyte derived epithelial-like Huh7 cell line	Approved as an immunosuppressive agent for the prophylaxis of organ rejection in patients (age ≥ 13)	Trials Progress	(82-84)
	**Nitazoxanide**	• Antiviral/parasitic drug<br>• Suppresses viral replication by inhibiting the maturation of the viral hemagglutinin and the viral transcription factor immediate early 2 (IE2), as well as by activating the eukaryotic translation initiation factor 2α (an antiviral intracellular protein)<br>• Inhibits the replication of several RNA/DNA viruses<br><br>Inhibits replication of SARS-CoV-2 *in vitro* in Vero E6 cell line	Approved for treatment of diarrhea caused by *Giardia lamblia* or *Cryptosporidium parvum* in non-immunosuppressed adults and children	Trials Progress	(68, 85)
	**Ivermectin**	• Antiparasitic drug shown to have antiviral activity *in vitro*<br>• Potentially inhibits IMPα/β1-mediated nuclear import of viral proteins, including those of SARS-CoV<br><br>Inhibits replication of SARS-CoV-2 *in vitro* in Vero/hSLAM cell line	Approved for the treatment of strongyloidiasis of the intestinal tract, and of Onchocerciasis	Trials Progress	(86)

COVID-19 progression	Drug name/Therapeutics	Mechanism of action/Potential role in COVID-19	Drug status	Clinical trials	References
**Mild COVID-19 phase (pre-pulmonary damage)**<br><br>**(See also Figure 1)**	**Ciclesonide**	• Glucocorticoid receptor agonist that can inhibit leukocyte infiltration at the inflammation site, interfere with inflammatory response mediators and suppress humoral immune responses<br>• Reduces the inflammatory reaction by limiting the capillary dilation and the permeability of the vascular structures<br><br>The use of inhaled ciclesonide may improve the outcome of COVID-19 patients	Approved for the treatment of asthma in patients 12 years of age or older	Trial Progress	(87)
	**Type I Interferon (IFN)**	• IFNα/β exert immunomodulatory antiviral effects by inhibiting viral protein synthesis, inactivating viral RNA and enhancing phagocytic and cytotoxic mechanisms against the virus<br><br>Type I IFN response evasion strategies have been described for SARS-CoV and MERS-CoV, and could be shared by SARS-CoV-2. Administration of type I IFNs may enhance antiviral immunity against SARS-CoV-2 if given early, but it may also promote hyperinflammation	Approved for:<br>• HCV and some HBV infections of patients with compensated liver disease<br>• Venereal or genital warts caused by HPV, or oral warts arising from HIV infection<br>• Hairy cell leukemia, malignant melanoma and AIDS-related Kaposi's sarcoma<br>• External condylomata acuminata<br>• Relapsing forms of MS	Trial Progress	(29, 88)
**Moderate and Severe COVID-19 phases (Hyperinflammation)**<br><br>**(See also Figure 2)**	**Convalescent Plasma Transfer**	• Plasma-therapy consisting in a pool of antiviral polyclonal hyperimmune globulin from recovered donors<br>• May suppress viremia<br>• May increase anti-virus neutralizing antibodies<br><br>Appears to improve clinical parameters in severe COVID-19 patients, including oxyhemoglobin saturation, lung lesions, levels of C-reactive protein, viremia, and lymphocyte counts. Appears to reduce hospitalization time and mortality. Plasma transfer could be more beneficial when viral titers are low	Not FDA Approved	Trial Progress	(89-91)
	**Complement Inhibitors**	• AMY-101 binds to complement component C3, inhibiting its cleavage into C3a and C3b<br>• Eculizumab is a humanized monoclonal antibody against complement C5. It prevents the cleavage of C5 into C5a and C5b, inhibiting deployment of the terminal complement system including the formation of the membrane attack complex<br><br>Blockage of complement components can prevent ARDS and reduce systemic inflammation. Therefore, treatments with complement inhibitors could prevent development of severe complications in COVID-19	Eculizumab is approved for:<br>• Paroxysmal nocturnal hemoglobinuria<br>• Atypical hemolytic uremic syndrome<br>• Neuromyelitis optica spectrum disorder	No registered trials to date with AMY-101<br><br>Trial Progress (with Eculizumab)	(48, 50, 92)
	**Canakinumab**	• Recombinant human anti-human IL-1β mAb<br>• Neutralizes the activity of the pro-inflammatory cytokine IL-1β by blocking its interaction with specific receptors<br><br>Since IL-1β has been found elevated in the plasma of severely ill, SARS-CoV-2 infected patients, administration of canakinumab could block IL-1-driven hyperinflammation and improve the outcome of COVID-19	Approved for autoinflammatory periodic fever syndromes, active systemic juvenile idiopathic arthritis and cryopyrin-associated periodic syndrome	Trial Progress	(7, 17)
	**Anakinra**	• Recombinant non-glycosylated form of the human IL-1R antagonist that blocks the activity of IL-1<br><br>Administration of anakinra could block IL-1-driven hyperinflammation and improve the outcome of COVID-19	Approved for rheumatoid arthritis and cryopyrin-associated periodic syndrome	Trial Progress	(7, 17)

COVID-19 progression	Drug name/Therapeutics	Mechanism of action/Potential role in COVID-19	Drug status	Clinical trials	References
**Moderate and Severe COVID-19 phases (Hyperinflammation)**<br><br>**(See also Figure 2)**	**Colchicine**	• Disrupts microtubules by binding tubulin, interfering with intracellular trafficking, cytokine and chemokine secretion, cell migration, and regulation of ion channels and cell division<br>• Counteracts the assembly of the NLRP3 inflammasome, limiting the release of IL-1β, and other cytokines like IL-6<br><br>SARS-CoV viroporins can activate the NLRP3 inflammasome by forming Ca2C-permeable ion channels. Potential interference with the secretion of IL-1β and other downstream cytokines by colchicine may limit hyperinflammation observed in severe COVID-19 patients due to cytokine release syndrome	Approved for gout and exacerbations of Familial Mediterranean Fever	Trial Progress	(7, 11, 93)
	**Tocilizumab**	• Recombinant humanized anti-human-IL-6 receptor mAb<br>• Binds to soluble/membrane-bound IL-6 receptors and inhibits IL-6-mediated signaling<br><br>Administration of tocilizumab may decrease SARS-CoV-2-driven hyperinflammation in COVID-19 patients with high levels of IL-6, by preventing macrophage and DC differentiation/activation, as well as T cell polarization towards Th2 and Th17 phenotypes. Of importance is the timing to apply the blocking therapy, considering the pleiotropic nature of IL-6	Approved for:<br>• Rheumatoid arthritis<br>• Giant cell arteritis<br>• Polyarticular and systemic juvenile idiopathic arthritis<br>• Cytokine release syndrome for patients undergoing CAR T cell therapy	Trial Progress	(11, 94-97)
	**Sarilumab**	• Recombinant fully human anti-IL-6 receptor mAb<br>• Binds to soluble/membrane-bound IL-6 receptors and inhibit IL-6-mediated signaling<br>• Has higher affinity for monomeric human and monkey IL-6, and a longer half-life than tocilizumab<br><br>Administration of sarilumab may decrease SARS-CoV-2 driven hyperinflammation in COVID-19 patients with high levels of IL-6, by preventing macrophage and DC differentiation/activation, as well as T cell polarization towards Th2 and Th17 phenotypes. Of importance is the timing to apply the blocking therapy, considering the pleiotropic nature of IL-6	Approved for moderately to severely active rheumatoid arthritis (RA)	Trial Progress	(11, 95, 97, 98)
	**Emapalumab**	• Recombinant fully human anti-human-IFN-γ mAb<br>• Suppresses hyperinflammation by binding and neutralizing IFN-γ in patients with hemophagocytic lymphohistiocytosis (HLH)<br><br>Improves clinical symptoms and survival in murine models of primary and secondary HLH by decreasing IFN-γ levels driven by virus infection or TLR activation, respectively. May decrease SARS-CoV-2-driven hyperinflammation in COVID-19 patients with high levels of IFN-γ, by blocking immune cell activation	Approved for adult and pediatric primary hemophagocytic lymphohistiocytosis (HLH)	Trial Progress	(7, 99)
	**Ruxolitinib**	• Acts as an immunomodulator by selectively inhibiting JAK1/2. JAK1 is involved in the signaling pathways of several pro-inflammatory cytokines including IL-2, IL-15, IL-6, IFN-γ and TNF<br>• Acts on cells of the innate and adaptive immune system, inhibiting NK cell function, macrophage cytokine release, and DC maturation, migration and function. In addition, decreases Treg counts and T helper cell cytokine secretion.<br><br>Could reduce the hyperinflammation observed in COVID-19 patients and thus prevent damage to the lung and other organs	Approved for intermediate or high-risk myelofibrosis	Trial Progress	(7, 11, 100)

COVID-19 progression	Drug name/Therapeutics	Mechanism of action/Potential role in COVID-19	Drug status	Clinical trials	References
**Moderate and Severe COVID-19 phases (Hyperinflammation)**<br><br>**(See also Figure 2)**	**Secukinumab**	• Recombinant human anti-IL-17A mAb<br>• Selectively binds to IL-17A and inhibits its interaction with the IL-17 receptor<br><br>Anti-IL-17A mAb treatment reduced inflammation due to cytokine release and oxidative stress in acute lung injury mouse models. Elevated levels of IL-17 were detected in ARDS patients. Secukinumab could reduce hyperinflammation driven by Th17 cells in severe COVID-19 patients with ARDS	Approved for adults with moderate to severe plaque psoriasis, active psoriatic arthritis, and ankylosing spondylitis	No registered trials to date	(4, 101, 102)
	**Ixekizumab**	• Recombinant humanized anti-IL-17A mAb<br>• Selectively binds to IL-17A and inhibits its interaction with the IL-17 receptor<br><br>Anti-IL-17A mAb treatment reduced inflammation due to cytokine release and oxidative stress in acute lung injury mouse models. Elevated levels of IL-17 were detected in ARDS patients. Ixekizumab could reduce hyperinflammation driven by Th17 cells in severe COVID-19 patients with ARDS	Approved for adults with moderate to severe plaque psoriasis, active psoriatic arthritis, and ankylosing spondylitis	No registered trials to date	(4, 101, 102)
	**Brodalumab**	• Recombinant fully human anti-IL-17 receptor A (IL-17RA) mAb<br>• Selectively binds to IL-17RA and inhibits its interactions with cytokines IL-17A, IL-17F, IL-17C, IL-17A/F heterodimer and IL-25<br><br>Elevated levels of IL-17 were detected in ARDS patients. Brodalumab could reduce hyperinflammation driven by Th17 cells in severe COVID-19 patients with ARDS	Approved for adults with moderate-to-severe plaque psoriasis	No registered trials to date	(4, 102, 103)
	**Methylprednisolone**	• Potent anti-inflammatory glucocorticoid<br><br>Although glucocorticoids are not recommended during the early stage of viral infection because they may impair antiviral immunity and delay virus clearance, methylprednisolone given in low doses may reduce hyperinflammation during the moderate to severe stage of COVID-19. Preliminary clinical data indicate that methylprednisolone may reduce the risk of death among severe COVID-19 patients with ARDS.	Approved to treat:<br>• Endocrine disorders<br>• Rheumatic disorders<br>• Collagen diseases<br>• Dermatologic diseases<br>• Allergic states<br>• Ophthalmologic diseases<br>• Respiratory diseases<br>• Hematologic disorders<br>• Neoplastic disease<br>• Edematous states<br>• Gastrointestinal diseases<br>• Acute exacerbations of MS	Trials Progress	(11, 17)
	**Nitric Oxide**	• When inhaled, nitric oxide (NO) selectively dilates the pulmonary vasculature, with minimal effect on the systemic vasculature<br>• Attenuates leukocyte activation and inflammatory responses, reduces platelet aggregation, has a bronchodilator effect, and facilitates the production of surfactant<br><br>NO inhibits viral replication of SARS-CoV in Vero E6 cell line	Approved for neonates with hypoxic respiratory failure (associated with clinical or echocardiographic evidence of pulmonary hypertension in conjunction with ventilatory support and other appropriate agents)	Trial Progress	(104, 105)

COVID-19 progression	Drug name/Therapeutics	Mechanism of action/Potential role in COVID-19	Drug status	Clinical trials	References
**Severe COVID-19 phase (Hyperinflammation)**<br><br>**(See also Figure 2)**	**Mesenchymal stem cell (MSC)**	• Stem cell therapy/Immunomodulator<br><br>Diminished hyperinflammation in COVID-19 patients by decreasing the levels of pro-inflammatory cytokines and hyperreactive T and NK cells, increasing Treg cells, and restoring peripheral blood lymphocyte levels. Infused MSC did not expressed ACE2	Not FDA Approved	Trial Progress	(106, 107)
	**Hyaluronidase**	• Enzyme that catalyzes the degradation of hyaluronan (HA)<br><br>Could decrease elevated HA, observed in the lungs of patients with ARDS, restoring lung function, as demonstrated in a mouse model of severe lung injury. May relieve pulmonary damage by decreasing fluid accumulation in the lungs of COVID-19 patients with ARDS	Approved as an adjuvant in subcutaneous fluid administration for achieving hydration and for increasing the absorption and dispersion of other injected drugs	No registered trials to date	(54, 108-110)
	**Hymecromone**	• Inhibits HA synthase by depleting the cell of its substrate, uridine-diphosphate-glucuronic acid and by downmodulating the expression of HAS2 and HAS3 mRNA<br><br>Reduced pulmonary hypertension and inflammation in mouse models of lung injury by decreasing the levels of HA. May relieve pulmonary damage in COVID-19 patients by decreasing HA accumulation in the lungs	Not FDA Approved<br><br>Approved in Europe and Asia to treat biliary spasm		

REFERENCES

61. O'Neil LAJ and Netea MG. BCG-induced trained immunity: can it offer protection against COVID-19? *Nat Rev Immunol.* (2020). doi: 10.1038/s41577-020-0337-y. [Epub ahead of print].

62. de Bree LCJ, Koeken VACM, Joosten LAB, Aaby P, Benn CS, van Crevel R, et al. Non-specific effects of vaccines: current evidence and potential implications. *Semin Immunol.* (2018) 39:35–43. doi: 10.1016/j.smim.2018.06.002

63. Yamamoto M, Matsuyama S, Li X, Takeda M, Kawaguchi Y, Inoue J-i, et al. Identification of nafamostat as a potent inhibitor of middle east respiratory syndrome coronavirus s protein-mediated membrane fusion using the split-protein-based cell-cell fusion assay. *Antimicrob Agents Chemother.* (2016) 60:6532–9. doi: 10.1128/aac.01043-16

64. Monteil V, Kwon H, Prado P, Hagelkrüys A, Wimmer RA, Stahl M, et al. Inhibition of SARS-CoV-2 infections in engineered human tissues using clinical-grade soluble human ACE2. *Cell.* (2020) 181:1–9. doi: 10.1016/j.cell.2020.04.004

65. Fang L, Karakiulakis G, Roth M. Are patients with hypertension and diabetes mellitus at increased risk for COVID-19 infection? *Lancet Respir Med.* (2020) 8:e21. doi: 10.1016/S2213-2600(20)30116-8

66. Sommerstein R, Kochen MM, Messerli FH, Gräni C. Coronavirus Disease 2019 (COVID-2019): Do angiotensin-converting enzyme inhibitors/angiotensin receptor blockers have a biphasic effect? *J Am Heart Assoc.* (2020) 9:e016509. doi: 10.1161/JAHA.120.016509

67. Patel AB, Verma A. COVID-19 and Angiotensin-converting enzyme inhibitors and angiotensin receptor blockers: what is the evidence? *JAMA.* (2020) 323:1769–70. doi: 10.1001/jama.2020.4812

68. Wang M, Cao R, Zhang L, Yang X, Liu J, Xu M, et al. Remdesivir and chloroquine effectively inhibit the recently emerged novel coronavirus (2019-nCoV) *in vitro*. *Cell Res.* (2020) 30:269–71. doi: 10.1038/s41422-020-0282-0

69. Devaux CA, Rolain J-M, Colson P, Raoult D. New insights on the antiviral effects of chloroquine against coronavirus: what to expect for COVID-19? *Int J Antimicrob Agents.* (2020) 105938. doi: 10.1016/j.ijantimicag.2020.105938

70. Cortegiani A, Ingoglia G, Ippolito M, Giarratano A, Einav S. A systematic review on the efficacy and safety of chloroquine for the treatment of COVID-19. *J Critical Care.* (2020). doi: 10.1016/j.jcrc.2020.03.005. [Epub ahead of print].

71. Colson P, Rolain J-M, Lagier J-C, Brouqui P, Raoult D. Chloroquine and hydroxychloroquine as available weapons to fight COVID-19. *Int J Antimicrob Agents.* (2020) 55:105932. doi: 10.1016/j.ijantimicag.2020.105932

72. Yao X, Ye F, Zhang M, Cui C, Huang B, Niu P, et al. *In vitro* antiviral activity and projection of optimized dosing design of hydroxychloroquine for the treatment of severe acute respiratory syndrome coronavirus 2 (SARS-CoV-2). *Clin Infect Dis.* (2020) ciaa237. doi: 10.1093/cid/ciaa237

73. Pfefferle S, Schöpf J, Kögl M, Friedel CC, Müller MA, Carbajo-Lozoya J, et al. The SARS-Coronavirus-host interactome: identification of cyclophilins as target for pan-coronavirus inhibitors. *PLOS Pathog.* (2011) 7:e1002331. doi: 10.1371/journal.ppat.1002331

74. de Wilde AH, Zevenhoven-Dobbe JC, van der Meer Y, Thiel V, Narayanan K, Makino S, et al. Cyclosporin A inhibits the replication of diverse coronaviruses. *J Gen Virol.* (2011) 92:2542–48. doi: 10.1099/vir.0.034983-0

75. Tanaka Y, Sato Y, Sasaki T. Suppression of coronavirus replication by cyclophilin inhibitors. *Viruses.* (2013) 5:1250–60. doi: 10.3390/v5051250

76. Furuta Y, Komeno T, Nakamura T. Favipiravir (T-705), a broad spectrum inhibitor of viral RNA polymerase. *Proc Japan Acad Series B.* (2017) 93:449–63. doi: 10.2183/pjab.93.027

77. Agostini ML, Andres EL, Sims AC, Graham RL, Sheahan TP, Lu X, et al. Coronavirus susceptibility to the antiviral remdesivir (GS-5734) is mediated by the viral polymerase and the proofreading exoribonuclease. *mBio.* (2018) 9:e00221–18. doi: 10.1128/mBio.00221-18

78. Sheahan TP, Sims AC, Graham RL, Menachery VD, Gralinski LE, Case JB, et al. Broad-spectrum antiviral GS-5734 inhibits both epidemic and zoonotic coronaviruses. *Sci Transl Med.* (2017) 9:eaal3653. doi: 10.1126/scitranslmed.aal3653

79. Ko W-C, Rolain J-M, Lee N-Y, Chen P-L, Huang C-T, Lee P-I, et al. Arguments in favour of remdesivir for treating SARS-CoV-2 infections. *Int J Antimicrob Agents.* (2020) 55:105933. doi: 10.1016/j.ijantimicag.2020.105933

80. Grein J, Ohmagari N, Shin D, Diaz G, Asperges E, Castagna A, et al. Compassionate use of remdesivir for patients with severe Covid-19. *N Engl J Med.* (2020). doi: 10.1056/NEJMoa2007016 [Epub ahead of print].

81. Yin W, Mao C, Luan X, Shen D-D, Shen Q, Su H, et al. Structural basis for inhibition of the RNA-dependent RNA polymerase from SARS-CoV-2 by remdesivir. *Science.* (2020) eabc1560. doi: 10.1126/science.abc1560

82. Stöhr S, Costa R, Sandmann L, Westhaus S, Pfaender S, Anggakusuma, et al. Host cell mTORC1 is required for HCV RNA replication. *Gut.* (2016) 65:2017–28. doi: 10.1136/gutjnl-2014-308971

83. Kindrachuk J, Ork B, Hart BJ, Mazur S, Holbrook MR, Frieman MB, et al. Antiviral Potential of ERK/MAPK and PI3K/AKT/mTOR signaling modulation for middle east respiratory syndrome coronavirus infection as identified by temporal kinome analysis. *Antimicrob Agents Chemother.* (2015) 59:1088–99. doi: 10.1128/aac.03659-14

84. Wang C-H, Chung F-T, Lin S-M, Huang S-Y, Chou C-L, Lee K-Y, et al. Adjuvant treatment with a mammalian target of rapamycin inhibitor, sirolimus, and steroids improves outcomes in patients with severe H1N1 pneumonia and acute respiratory failure. *Critical Care Med.* (2014) 42:313–21. doi: 10.1097/CCM.0b013e3182a2727d

85. Rossignol J-F. Nitazoxanide: A first-in-class broad-spectrum antiviral agent. *Antiviral Res.* (2014) 110:94–103. doi: 10.1016/j.antiviral.2014.07.014

86. Caly L, Druce JD, Catton MG, Jans DA, Wagstaff KM. The FDA-approved drug ivermectin inhibits the replication of SARS-CoV-2 *in vitro*. *Antiviral Res.* (2020) 178:104787. doi: 10.1016/j.antiviral.2020.104787

87. Iwabuchi K, Yoshie K, Kurakami Y, Takahashi K, Kato Y, Morishima T. Therapeutic potential of ciclesonide inhalation for COVID-19 pneumonia: report of three cases. *J Infect Chemother.* (2020) 26:625-32. doi: 10.1016/j.jiac.2020.04.007

88. Sallard E, Lescure F-X, Yazdanpanah Y, Mentre F, Peiffer-Smadja N. Type 1 interferons as a potential treatment against COVID-19. *Antiviral Res.* (2020) 178:104791. doi: 10.1016/j.antiviral.2020.104791

89. Chen L, Xiong J, Bao L, Shi Y. Convalescent plasma as a potential therapy for COVID-19. *Lancet Infect Dis.* (2020) 20:398–400. doi: 10.1016/S1473-3099(20)30141-9

90. Shen C, Wang Z, Zhao F, Yang Y, Li J, Yuan J, et al. Treatment of 5 critically ill patients with COVID-19 with convalescent plasma. *JAMA.* (2020) 323:1582–89. doi: 10.1001/jama.2020.4783

91. Duan K, Liu B, Li C, Zhang H, Yu T, Qu J, et al. Effectiveness of convalescent plasma therapy in severe COVID-19 patients. *Proc Natl Acad Sci USA.* (2020) 117:9490–96. doi: 10.1073/pnas.2004168117

92. Diurno F, Numis FG, Porta G, Cirillo F, Maddaluno S, Ragozzino A, et al. Eculizumab treatment in patients with COVID-19: preliminary results from real life ASL napoli 2 nord experience. *Eur Rev Med Pharmacol Sci.* (2020) 24:4040–47. doi: 10.26355/eurrev_202004_20875

93. Chen I-Y, Moriyama M, Chang M-F, Ichinohe T. Severe acute respiratory syndrome coronavirus viroporin 3a activates the NLRP3 inflammasome. *Front Microbiol.* (2019) 10:1–9. doi: 10.3389/fmicb.2019.00050

94. Moore BJB, June CH. Cytokine release syndrome in severe COVID-19. *Science.* (2020) 368:473–74. doi: 10.1126/science.abb8925

95. Avci AB, Feist E, Burmester GR. Targeting IL-6 or IL-6 receptor in rheumatoid arthritis: what's the difference? *BioDrugs.* (2018) 32:531–46. doi: 10.1007/s40259-018-0320-3

96. Kotch C, Barrett D, Teachey DT. Tocilizumab for the treatment of chimeric antigen receptor T cell-induced cytokine release syndrome. *Expert Rev Clin Immunol.* (2019) 15:813–22. doi: 10.1080/1744666X.2019.1629904

97. Velazquez-Salinas L, Verdugo-Rodriguez A, Rodriguez LL, Borca MV. The role of interleukin 6 during viral infections. *Front Microbiol.* (2019) 10:1–6. doi: 10.3389/fmicb.2019.01057

98. Lamb YN, Deeks ED. Sarilumab: A review in moderate to severe rheumatoid arthritis. *Drugs.* (2018) 78:929–40. doi: 10.1007/s40265-018-0929-z

99. Vallurupalli M, Berliner N. Emapalumab for the treatment of relapsed/refractory hemophagocytic lymphohistiocytosis. *Blood.* (2019) 134:1783–6. doi: 10.1182/blood.2019002289

100. Elli EM, Baratè C, Mendicino F, Palandri F, Palumbo GA. Mechanisms underlying the anti-inflammatory and immunosuppressive activity of ruxolitinib. *Front Oncol.* (2019) 9:1186. doi: 10.3389/fonc.2019.01186

101. Righetti RF, dos Santos TM, do Nascimento Camargo L, Aristóteles LRCRB, Fukuzaki S, de Souza FCR, et al. Protective effects of anti-IL17 on acute lung injury induced by LPS in mice. *Front Pharmacol.* (2018) 9:1021. doi: 10.3389/fphar.2018.01021

102. Li Q, Gu Y, Tu Q, Wang K, Gu X, Ren T. Blockade of interleukin-17 restrains the development of acute lung injury. *Scand J Immunol.* (2016) 83:203–11. doi: 10.1111/sji.12408

103. Golbari NM, Basehore BM, Zito PM. Brodalumab. In: StatPearls. Treasure Island, FL: StatPearls Publishing LLC (2020). p. 1-20

104. Åkerström S, Mousavi-Jazi M, Klingström J, Leijon M, Lundkvist Å, Mirazimi A. Nitric oxide inhibits the replication cycle of severe acute respiratory syndrome coronavirus. *J Virol.* (2005) 79:1966–69. doi: 10.1128/JVI.79.3.1966-1969.2005

105. Papazian L, Aubron C, Brochard L, Chiche J-D, Combes A, Dreyfuss D, et al. Formal guidelines: management of acute respiratory distress syndrome. *Ann Intensive Care.* (2019) 9:69. doi: 10.1186/s13613-019-0540-9

106. Leng Z, Zhu R, Hou W, Feng Y, Yang Y, Han Q, et al. Transplantation of ACE2 negative mesenchymal stem cells improves the outcome of patients with COVID-19 pneumonia. *Aging Dis.* (2020) 11:216–28. doi: 10.14336/AD.2020.0228

107. Thanunchai M, Hongeng S, Thitithanyanont A. Mesenchymal stromal cells and viral infection. *Stem Cells Int.* (2015) 2015:1–8. doi: 10.1155/2015/860950

108. McKallip RJ, Hagele HF, Uchakina ON. Treatment with the hyaluronic acid synthesis inhibitor 4-methylumbelliferone suppresses SEB-induced lung inflammation. *Toxins.* (2013) 5:1814–26. doi: 10.3390/toxins5101814

109. Hällgren R, Samuelsson T, Laurent TC, Modig J. Accumulation of hyaluronan (hyaluronic acid) in the lung in adult respiratory distress syndrome. *Am Rev Respir Dis.* (1989) 139:682–7. doi: 10.1164/ajrccm/139.3.682

110. Collum SD, Chen N-Y, Hernandez AM, Hanmandlu A, Sweeney H, Mertens TCJ, et al. Inhibition of hyaluronan synthesis attenuates pulmonary hypertension associated with lung fibrosis. *Br J Pharmacol.* (2017) 174:3284–301. doi: 10.1111/bph.13947
